# Increased *MAGE-C* Family Gene Expression Levels as a Biomarker of Colon Cancer Through the Demethylation Mechanism

**DOI:** 10.3390/ph17111447

**Published:** 2024-10-29

**Authors:** Mikhlid H. Almutairi, Waad A. Alsoraie, Turki M. Alrubie, Ahmad S. Alkhaldi, Nada S. Alhajri, Monira A. Alaujan, Manar H. Almutairi, Bader O. Almutairi

**Affiliations:** 1 Zoology Department, College of Science, King Saud University, P.O. Box 2455, Riyadh 11451, Saudi Arabia; 442203429@student.ksu.edu.sa (W.A.A.); 443106654@student.ksu.edu.sa (A.S.A.); nada.alhajri44@gmail.com (N.S.A.); 443204206@student.ksu.edu.sa (M.A.A.); 445205486@student.ksu.edu.sa (M.H.A.); bomotairi@ksu.edu.sa (B.O.A.); 2 Laboratories Directorate, General Directorate of Animal Health, Ministry of Environment, Water and Agriculture, Riyadh 11195, Saudi Arabia; talrubie@mewa.gov.sa

**Keywords:** *MAGE-C1*, *MAGE-C2*, *MAGE-C3*, colon cancer, expression, 5-aza-2′-deoxycytidine

## Abstract

Background/Objectives: Colon cancer (CC) in Saudi Arabia is associated with a high death rate and is commonly identified at a more progressive stage. Therefore, it is critical to identify and characterize potential novel cancer-specific biomarkers to enhance early CC diagnosis. The goal was to assess their potential use as cancer biomarkers for the early detection and improvement of CC treatment. Methods: *MAGE-C1*, *MAGE-C2*, and *MAGE-C3* family gene expression levels were examined using RT-PCR and qRT-PCR assays in 26 adjacent normal colon (NC) and CC tissue samples from male and female Saudi patients. Using several cell lines and the qRT-PCR technique, epigenetic control was also investigated to determine whether reduced treatment with 5-aza-2′-deoxycytidine, which reduces DNA methyltransferase, can increase the expression of the *MAGE-C* gene. The expression levels, promoter methylation, and prognostic significance of MAGE-C1, MAGE-C2, and MAGE-C3 genes across various cancers were analyzed using The Cancer Genome Atlas (TCGA) data. Additionally, the prognostic significance of these genes was assessed through Kaplan–Meier survival analysis. Results: The RT-PCR results showed that *MAGE-C1*, *MAGE-C2*, and *MAGE-C3* gene expressions were significantly higher in the CC and NC tissues. The *MAGE-C1* expression level was the highest in CC tissues (*p* < 0.0001), followed by *MAGE-C3* (*p* = 0.0004) and *MAGE-C2* (*p* = 0.0020) in descending order. The 5-aza-2′-deoxycytidine treatment significantly increased the mRNA expression levels of the *MAGE-C1*, *MAGE-C2*, and *MAGE-C3* genes in HCT116, Caco-2, MCF-7, and MCF-10A cells. Expression analyses of TCGA samples revealed significant upregulation of these genes in several cancer types, with notable differences between normal, tumor, and metastatic tissues. Promoter methylation indicates hypomethylation in cancerous tissues. Survival analyses show that high expression levels of MAGE-C1 correlate with better prognosis, while MAGE-C3 is associated with poorer outcomes. Conclusions: These results demonstrate that *MAGE-C* genes are viable prospective biomarkers of CC controlled by hypomethylating drugs, consequently offering a possible treatment target for CC in a specific population.

## 1. Introduction

In Saudi Arabia, colon cancer (CC) ranks as one of the leading causes of cancer mortality among both genders. It is the most frequently diagnosed cancer in men and holds the third position in terms of diagnosis for women [1]. There are two main reasons for this high mortality and the prevalence rates among the Saudi population: the poor efficacy of existing treatments and the late detection of symptoms [2]. Additionally, CC is prevalent among both men and women in the 55-to-58 age range [3]. However, younger ages have shown a rising rate of CC prevalence in recent years in Saudi Arabia [4]. Recent estimates indicate that over 73% of CC cases in Saudi patients are identified at a late stage [5]. As a consequence of these investigations, identifying new possible cancer-specific biomarkers is essential to improve the early detection and diagnosis of CC [2,6].

Cancer-testis (CT) genes represent a unique class of genes that are normally expressed only in the human germline but become aberrantly activated in various cancers [7]. CT genes are only expressed in normal tissues, particularly in the adult testis; nonetheless, they are often aberrantly expressed in different types of cancers, including CC [7,8,9]. The CT genes are immune-privileged due to their lack or low expression of human leukocyte antigen molecules [10], also known as the human major histocompatibility complex (MHC). Consequently, the specificity and unique expressions of CT genes make them promising biomarkers for cancer diagnosis and attractive targets for immunotherapy in malignancies such as CC [7,11]. Testicular cells do not display MHC class I molecules, and the cytotoxic T lymphocytes do not recognize CT antigens [12]. Therefore, CT antigens are seen as excellent candidates for anticancer cell vaccines. We understand that the Ludwig Institute for Cancer Research has identified roughly 70 distinct CT gene families and more than 170 CT members on a free website (http://www.cta.lncc.br/, accessed on 1 February 2024) [13]. Multiple CT genes are also present in various normal tissues, including the pancreas, liver, spleen, and colon, but their expression levels in these tissues are significantly lower than those found in germ cells [1,7,14].

The melanoma-associated antigen subfamily C (MAGE-C) is a member of the CT gene family, which includes three known members of homologous genes arranged in serial order from *MAGE-C1* to *MAGE-C3* [15,16]. A previous study indicated that the expressions of *MAGE-C1* and *MAGE-C2* were associated with advanced breast cancer and may have potential targets for cancer immunotherapy [16]. *MAGE-C1*, the most frequently expressed gene in multiple myeloma (MM), was detected in both early and advanced diseases. Therefore, it was recognized as a diagnostic marker of MM disease [17]. Furthermore, the expression of *MAGE-C1* is linked to the progression of MM and resistance to apoptosis [18], and it has been identified as a significant predictive marker in melanoma [19] and ovarian cancer [20]. The *MAGE-C3* gene was overexpressed in esophageal squamous cell carcinoma tissues [21].

Studies have indicated that epigenetic processes, such as DNA methylation, play a significant role in regulating the expression of numerous CT genes [6,7,11,22]. DNA methyltransferase enzymes (DNMTs) suppress the expression of various CT genes by adding multiple methyl groups to their promoter regions, a process known as DNA hypermethylation [11,22]. Alternatively, DNA hypomethylation of various CT genes can be experimentally triggered by using DNA methyltransferase inhibitors (DNMTi) like 5-aza-2′-deoxycytidine, which removes methyl groups and enhances CT gene expression [6,7]. Previous studies have demonstrated that the application of 5-aza-2′-deoxycytidine to CC cell lines leads to the upregulation of several CT genes, including *MAGE-A1*, *MAGE-A3*, *MAGE-A4*, *MAGE-B1*, *SSX2*, *SCP2D1*, and *CTAG1A* [1,6,23]. While the expression of *MAGE-C* genes has been implicated in various cancers, the influence of epigenetic mechanisms on their regulation remains underexplored.

The aim of this study was to examine the expression profiles of the *MAGE-C* family genes in CC tissues from Saudi patients and to explore their potential as biomarkers for early detection of CC. The expression of these genes was evaluated in 26 samples of CC and matched normal colon (NC) tissue using RT-PCR and qRT-PCR assays. Additionally, this study examined the epigenetic regulation of *MAGE-C* gene expression by evaluating the effects of 5-aza-2′-deoxycytidine, a DNA methyltransferase inhibitor, on the transcriptional activation of *MAGE-C* genes in four different cell lines. The selection of the MAGE-C family for this investigation was based on two primary factors: firstly, MAGE-C family genes were sourced from the cancer-testis antigen (CTA) database due to their established expression in various cancers and their limited or absent expression in normal tissues (CTA database: http://www.cta.lncc.br/index.php (accessed on 1 February 2024). Secondly, *MAGE-C* gene expressions were observed in CC tissues or cell lines [8,24] and are linked to other cancers [16,17,20,21].

## 2. Results

### 2.1. Basic Clinical Characteristics of Study Subjects

A total of 26 samples of NC and CC tissues were analyzed, including 20 samples from males and 6 samples from females. Our research into the demographics of the donors found that the median age of male patients with CC was 57 years, while for female patients it was 59 years. Among male patients, 40% were younger than 57, while 60% were older than that same age. For female patients, the age distribution was evenly split, with 50% younger than 59 and 50% older. Table 1 lists additional clinical data of the individuals, such as their cancer stages.

### 2.2. Expression Profiles of the MAGE-C1, MAGE-C2, and MAGE-C3 Genes in the Matched CC and NC Tissues

**Figure 1:**
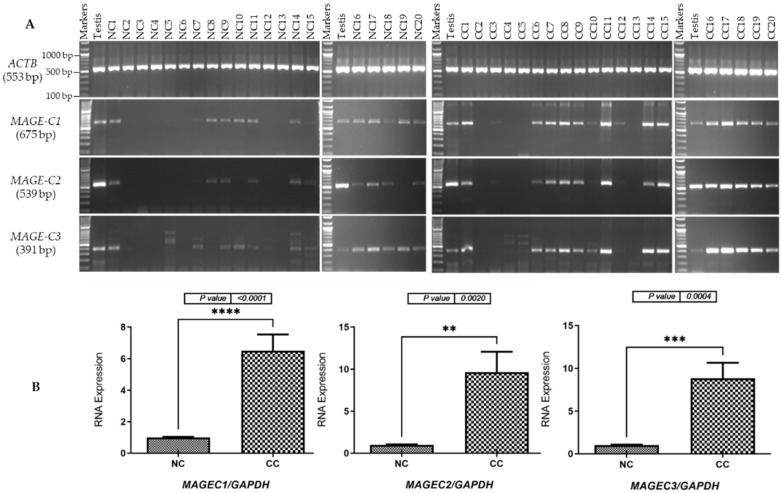
Identification of *MAGE-C* gene transcripts in tissue specimens collected from male patients with adjacent NC and CC tissues. (**A**) The RT-PCR results for the *MAGE-C* family genes are shown on agarose gel pictures. The cDNAs were created from the total RNA taken from 20 tissue samples, 20 of which were NC and 20 of which were CC. To ensure the integrity of the cDNA samples, the expression of the *ACTB* gene was evaluated, yielding a band of approximately 553 bp. The primers for each gene were validated using cDNA obtained from testicular tissue. The official names of the individual genes along with their expected product sizes are shown to the left of the gel images. (**B**) qRT-PCR analysis of *MAGE-C* family gene expression was measured in NC and CC samples. This qRT-PCR quantified the mRNA expression of these genes in CC samples relative to their respective NC samples, with results normalized to *GAPDH* mRNA levels. The error bars in the figure indicate the standard error of the mean from three separate qRT-PCR experiments for each gene. Significance is indicated by ** *p* ≤ 0.01, *** *p* ≤ 0.001, and **** *p* ≤ 0.0001. Abbreviations: NC (normal colon); CC (colon cancer).

**Figure 2:**
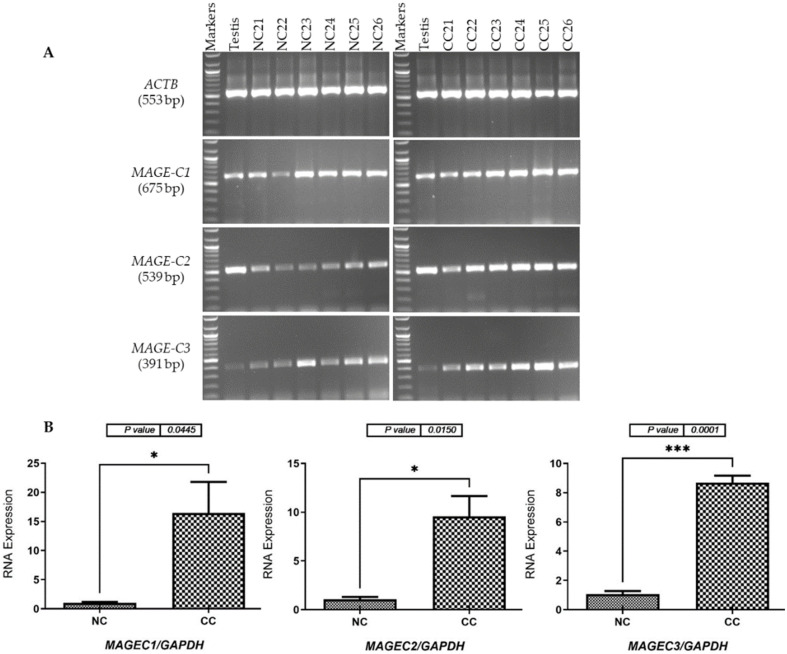
Identification of *MAGE-C* gene transcripts in tissue specimens collected from female patients with adjacent NC and CC tissues. Panel (**A**) displays agarose gel images depicting the results of RT-PCR performed for *MAGE-C1*, *MAGE-C2*, and *MAGE-C3*, using cDNAs synthesized from the total RNA extracted from six NC and six CC tissue samples. Panel (**B**) describes a qRT-PCR analysis that measured the expression levels of *MAGE-C* family genes in both NC and CC samples. This qRT-PCR quantified the mRNA expression of these genes in CC samples relative to their respective NC samples, with results normalized to *GAPDH* mRNA levels. The error bars in the figure indicate the standard error of the mean from three separate qRT-PCR experiments for each gene. Significance is indicated by * *p* ≤ 0.05 and *** *p* ≤ 0.001. Abbreviations: NC (normal colon); CC (colon cancer).

The expression levels of mRNA for the *MAGE-C* family were assessed by optimizing the primers and annealing temperatures to ensure the amplification of specific products for each gene. Subsequently, RT-PCR analysis was conducted to validate the expression of *MAGE-C* family genes, confirming the specificity of the results for testis tissue. To achieve this, various tissue samples were collected, including twenty NC samples from Saudi males (Figure 1A, left panel) and six NC samples from Saudi females (Figure 2A, left panel). cDNA synthesized from the total RNA of a human testis was used to validate the primers for each gene under investigation. The integrity of the cDNA obtained from both NC and CC samples was verified through the expression analysis of the *ACTB* gene. The RT-PCR results demonstrated increased expression levels of *MAGE-C1*, *MAGE-C2*, and *MAGE-C3* in CC tissue samples from both male and female patients (Figure 1A and Figure 2A, right panels), compared to the expression levels in NC tissue specimens (Figure 1A and Figure 2A, left panels).

We employed qRT-PCR to quantify the mRNA levels of *MAGE-C* family genes in CC and NC tissue samples from male and female participants. The qRT-PCR findings, illustrated in Figure 1B and Figure 2B, indicated that *MAGE-C1*, *MAGE-C2*, and *MAGE-C3* were significantly more highly expressed in CC tissues compared to NC tissues. Therefore, the results from the RT-PCR were consistent with the qRT-PCR analyses. In male patients, the expression levels of *MAGE-C1* in CC tissues were the highest (*p* < 0.0001), followed by *MAGE-C3* (*p* = 0.0004) and *MAGE-C2* (*p* = 0.0020), as shown in Figure 1B. For female patients, *MAGE-C3* exhibited the highest expression levels in CC tissues (*p* = 0.0001), while *MAGE-C2* followed with the second highest (*p* = 0.0150), and *MAGE-C1* ranked third (*p* = 0.0445), as shown in Figure 2B).

### 2.3. Detection of the DNMT1 Gene Expression in CC, Breast Cancer (BC), and Normal Breast (NB) Cell Lines

**Figure 3:**
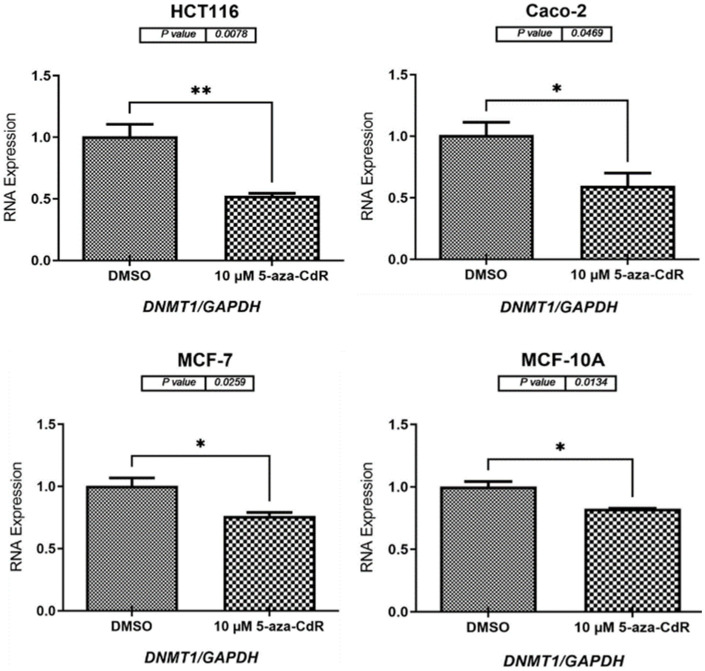
*DNMT1* expression data from qRT-PCR in HCT116, Caco-2, MCF-7, and MCF-10A cells following a 72 h treatment with 10 µM 5-aza-2′-CdR. The bar charts depict *DNMT1* expression in these cell lines both prior to and following the treatment, with control groups receiving DMSO, the treatment solvent. All expression data were normalized to *GAPDH* mRNA levels. Significance is indicated by * *p* ≤ 0.05 and ** *p* ≤ 0.01.

DNMT1 serves as the primary enzyme responsible for DNA methylation maintenance, and the administration of 5-aza-2′-deoxycytidine (5-aza-2′-CdR) has been revealed to decrease the expression of *DNMT1*. To investigate this relationship, we conducted an experiment to verify the hypothesis. We compared the expression levels of *DNMT1* across four human cell lines derived from different tissues—HCT116, Caco-2, MCF-7, and MCF-10A—using quantitative reverse transcription PCR (qRT-PCR) for analysis. As illustrated in Figure 3, treatment with 5-aza-2′-CdR resulted in a significant decrease in *DNMT1* expression levels in HCT116 (*p* = 0.0078), Caco-2 (*p* = 0.0469), MCF-7 (*p* = 0.0259), and MCF-10A (*p* = 0.0134) cells when compared to the DMSO-treated controls.

### 2.4. The Impact of 5-Aza-2′-CdR on the Morphology of the Cancer and Normal Cell Lines

The morphological changes in all cell lines were observed before and following 5-aza-2′-CdR treatment using an inverted microscope. The 5-aza-2′-CdR treatment reduced the proliferation of the treated cells (Table 2); however, no abnormalities were noted in the treated cells.

### 2.5. Effects of 5-Aza-2′-CdR on MAGE-C Gene Expressions in Various Types of Cell Lines

The hypomethylating drug has the potential to raise the levels of expression of a number of CT genes that have been discovered to this point [6,11]. Most of these genes are classified as X-CT genes, which require DNA sequence hypermethylation for their silencing. As a result, we conducted an investigation to determine whether the expressions of *MAGE-C* genes might be altered through the administration of 5-aza-2′-CdR. Additionally, we investigated whether the methylation process may potentially affect the expressions of certain *MAGE-C* genes in BC tissue samples. Cells that were treated with 5-aza-2′-CdR had their levels of mRNA measured, and the results were compared to those of cells that were treated with DMSO. In order to determine the potential effects on gene expression, a volume of 10 µL of DMSO was added to the cells. This was carried out because DMSO was the solvent that was utilized to dissolve the 5-aza-2′-CdR medication. After that, the mean and standard deviation of each gene expression level were computed for each individual sample.

**Figure 4:**
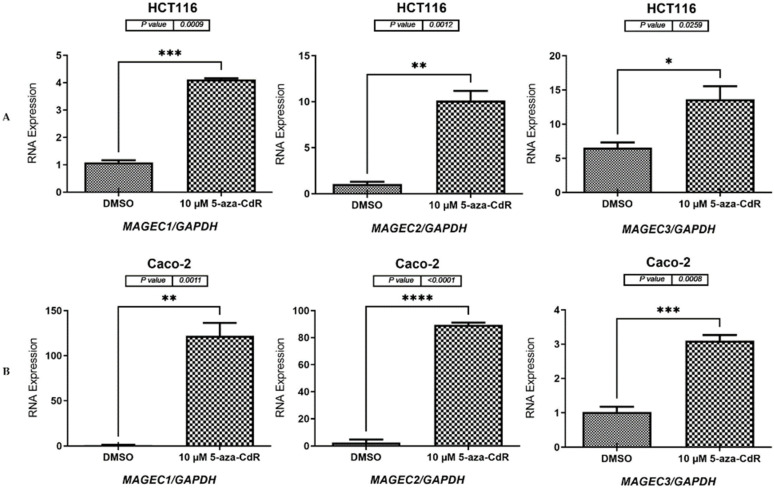
qRT-PCR results for *MAGE-C1*, *MAGE-C2*, and *MAGE-C3* in HCT116 and Caco-2 cells following a 72 h treatment with 10 µM of 5-aza-2′-CdR. (**A**,**B**) *MAGE-C1*, *MAGE-C2*, and *MAGE-C3* were analyzed using qRT-PCR in HCT116 (**A**) and Caco-2 (**B**) cells. *MAGE-C1*, *MAGE-C2*, and *MAGE-C3* expression levels in HCT116 and Caco-2 cells are shown in the bar charts before and after the 5-aza-2′-CdR treatment. DMSO was employed to treat the Caco-2 and HCT116 control cells because it was the solvent used to make the 5-aza-2′-CdR solution. The *GAPDH* mRNA levels were used as the reference point for normalizing the expression levels. The error bars depicted in the graphs indicate the standard error of the mean, which is calculated from three independent qRT-PCR experiments conducted for each gene. Significance levels are represented by the corresponding symbols: * *p* ≤ 0.05, ** *p* ≤ 0.01, *** *p* ≤ 0.001, and **** *p* ≤ 0.0001.

**Figure 5:**
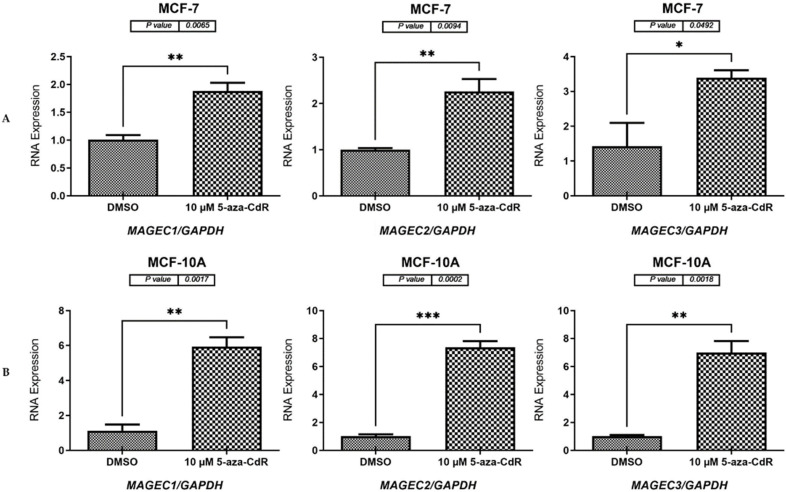
qRT-PCR results for *MAGE-C1*, *MAGE-C2*, and *MAGE-C3* in MCF-7 and MCF-10A cells following a 72 h treatment with 10 µM of 5-aza-2′-CdR. (**A**,**B**) *MAGE-C1*, *MAGE-C2*, and *MAGE-C3* were analyzed using qRT-PCR in MCF-7 (**A**) and MCF-10A (**B**) cells, respectively. *MAGE-C1*, *MAGE-C2*, and *MAGE-C3* expression levels in MCF-7 and MCF-10A cells are shown in the bar charts before and after the 5-aza-2′-CdR treatment. DMSO treated the MCF-7 and MCF-10A control cells because it was the solvent used to make the 5-aza-2′-CdR solution. The *GAPDH* mRNA levels were used as the reference point for normalizing the expression levels. The error bars depicted in the graphs indicate the standard error of the mean, which is calculated from three independent qRT-PCR experiments conducted for each gene. Significance levels are represented by the corresponding symbols: * *p* ≤ 0.05, ** *p* ≤ 0.01, and *** *p* ≤ 0.001.

To determine whether decreased DNA methyltransferase activity could stimulate the expression of *MAGE-C1*, *MAGE-C2*, and *MAGE-C3* genes, 10 µM 5-aza-2′-CdR was applied to HCT116, Caco-2, MCF-7, and MCF-10A cell lines for 72 h, as described in Section 2.2 and Section 2.3. Following the treatment, cDNA was created, and qRT-PCR was carried out, as detailed in Section 4.5 and Section 4.7. The qRT-PCR results for the CC cell lines (HCT116 and Caco-2), the BC cell line (MCF-7), and the NB cell (MCF-10A) demonstrated that cells treated with 5-aza-CdR exhibited significantly higher mRNA expression levels of the *MAGE-C1*, *MAGE-C2*, and *MAGE-C3* genes than the cells treated with DMSO. The specific *p*-values are as follows: HCT116 (*p* = 0.0009, *p* = 0.0012, and *p* = 0.0259, respectively, as shown in Figure 4A), Caco-2 (*p* = 0.0011, *p* < 0.0001, and *p* = 0.0008, respectively, as shown in Figure 4B), MCF-7 (*p* = 0.0065, *p* = 0.0094, and *p* = 0.0492, respectively, as shown in Figure 5A), and MCF-10A (*p* = 0.0017, *p* = 0.0002, and *p* = 0.0018, respectively, as shown in Figure 5B).

### 2.6. In Silico Analysis of MAGE-C Protein Family

**Figure 6:**
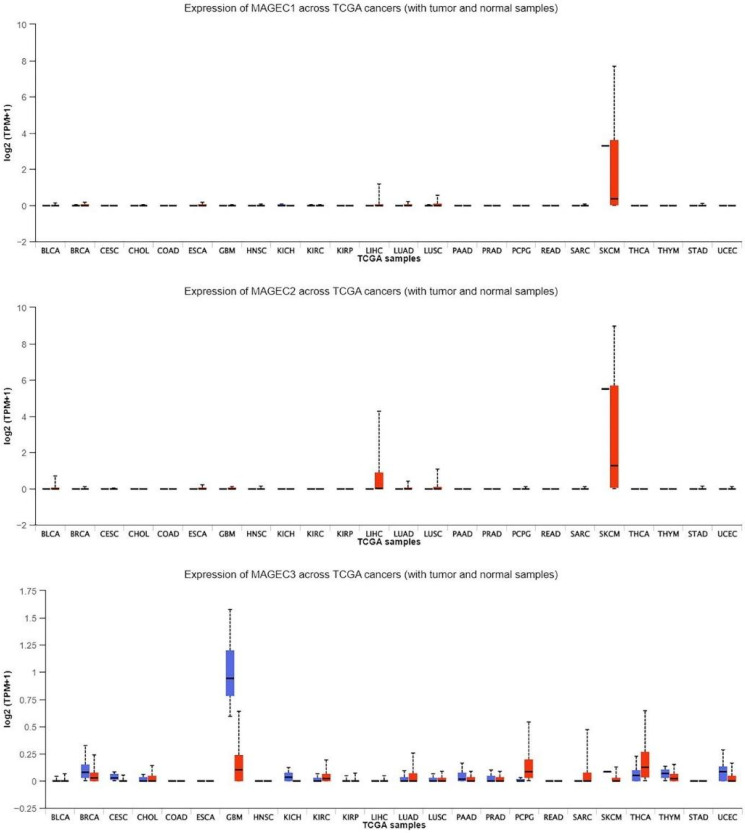
The expression levels of the genes MAGE-C1, MAGE-C2, and MAGE-C3 across various cancer types as part of the TCGA project. On the *y*-axis, the log2-transformed expression levels of these genes are presented in Transcripts Per Million (TPM), while the *x*-axis displays the abbreviations for different cancer types. The red bars represent different cancer tissues, while the blue bars correspond to different normal tissues.

**Figure 7:**
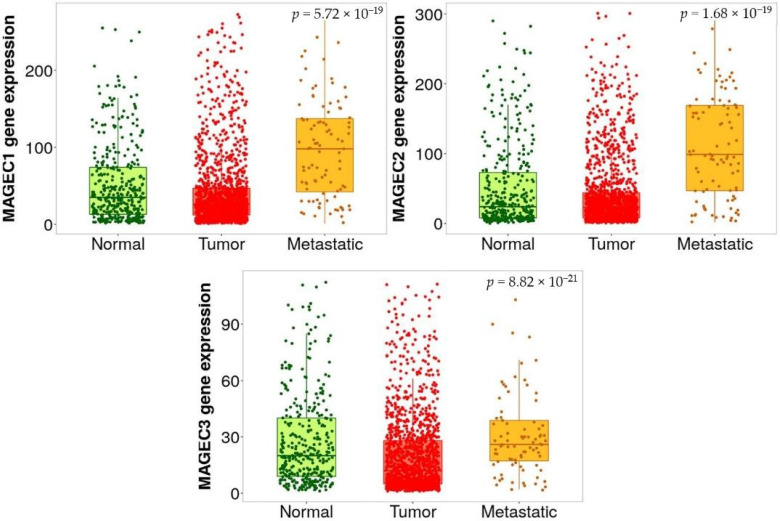
The expression levels of three MAGE-C genes are increased in both cancerous and metastatic tissues. This figure presents three box plots, each illustrating the expression levels of these genes across three different conditions: Normal, Tumor, and Metastatic tissues.

The mRNA expression of the three MAGE-C genes were examined using data from The Cancer Genome Atlas (TCGA) through the UALCAN database. This investigation encompassed samples from cancer tissues (*n* = 313) and normal tissues (*n* = 37) linked to three MAGE-C genes. The analysis revealed that the expression of three MAGE-C genes in normal tissues was low and did not achieve statistical significance, as shown in Figure 6. Although MAGE-C1 and MAGE-C2 exhibited a significant reduction in expression, the observed differences were not statistically significant (*p* > 0.05). Additionally, MAGE-C3 showed varying expression levels across different cancer types, with significantly higher expression in glioblastoma multiforme (GBM), Head and Neck Squamous Cell Carcinoma (HNSC), and Thyroid Carcinoma (THCA), as well as moderate levels in several other malignancies. Conversely, Figure 7 illustrated that three MAGE-C genes’ expressions were significantly higher in tumor and metastatic tissues compared to normal tissues, with all changes reaching high statistical significance. Specifically, MAGE-C1 and MAGE-C2 were expressed at much higher levels in metastatic tissues than tumor tissues, suggesting their potential role in metastasis. MAGE-C3, while having marginally lower expression in metastatic tissues compared to tumor tissues, still showed a significant increase compared to normal tissues. Thus, Figure 7 clarifies the genes’ roles in cancer development and metastasis, revealing significant upregulation in both tumor and metastatic tissues.

**Figure 8:**
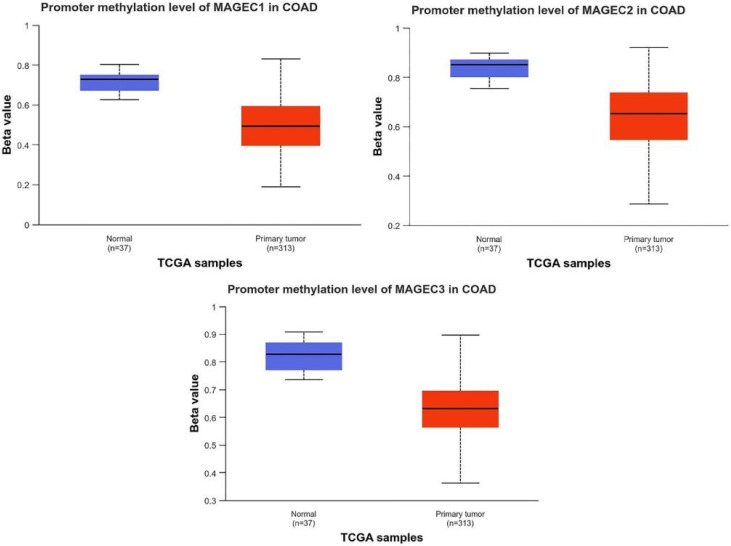
The promoter methylation levels of the genes *MAGE-C1*, *MAGE-C2*, and *MAGE-C3* in colorectal adenocarcinoma (COAD) samples, comparing normal tissue samples to primary tumor samples. The figure suggests that *MAGE-C* genes are hypomethylated in COAD tumor samples compared to normal tissues.

The DNA methylation status was confirmed using UALCAN. The three MAGE-C genes were identified as elevated hub genes with reduced methylation in their promoters. The predictive value of 20 methylation hub genes that were differentially expressed was analyzed by searching the Kaplan–Meier plotter database, specifically focusing on CC. The three MAGE-C genes show increased expression and decreased methylation levels (Figure 8). The promoter regions of all three genes (MAGE-C1, MAGE-C2, and MAGE-C3) exhibit lower methylation levels in primary tumor samples compared to normal samples. Hypomethylation, which refers to a decrease in methylation, is a frequent occurrence in cancer and is generally linked to the activation of gene expression. Reduced methylation in the promoter region can result in enhanced gene expression. This observation is consistent with prior data, indicating that the expression levels of these genes are higher in tumor and metastatic tissues compared to normal tissues (Figure 8).

### 2.7. Survival Analysis of MAGE-C Genes in a Kaplan–Meier Plotter

**Figure 9:**
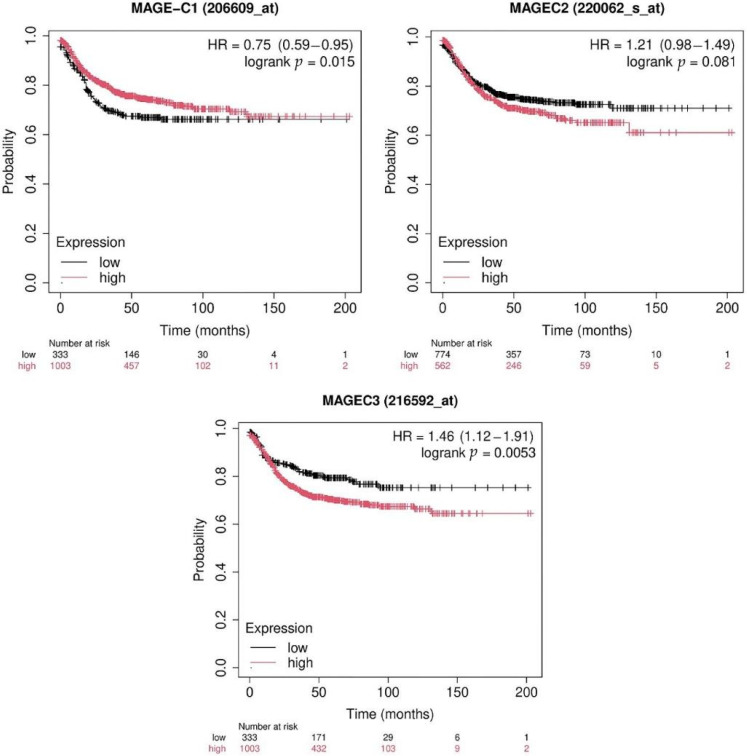
Kaplan–Meier survival plots, each showing the association between the expression levels of the genes MAGE-C1, MAGE-C2, and MAGE-C3 and the overall survival in a cohort of patients. Patients with a high expression (red curve) have worse survival than those with a low expression (black curve).

The survival analysis of MAGE-C family genes was performed using a Kaplan–Meier plotter. Patients exhibiting elevated levels of MAGE-C1 demonstrate much-improved survival rates compared to those with lower levels. This is evident from the higher survival curve, lower hazard ratio (HR = 0.75), and statistically significant log-rank *p*-value (0.015). This indicates that elevated MAGE-C1 expression may be linked to a more positive outcome. There is a 21% increase in mortality risk for patients with high MAGE-C2 expression compared to low expression (Figure 9). Patients who exhibit elevated levels of MAGE-C2 have a greater likelihood of mortality than those with lower levels, as seen by the hazard ratio (HR = 1.21).

Nevertheless, the disparity in survival rates between the two groups does not meet the criteria for statistical significance, as indicated by the log-rank p-value (0.081). These findings suggest that the expression of MAGE-C2 alone may not be a dependable indicator of patient survival in this situation. Patients with a high expression of MAGE-C3 have a significantly higher risk of death compared to those with a low expression, as indicated by the higher hazard ratio (HR = 1.46) and the statistically significant log-rank *p*-value (0.0053). This suggests that high MAGE-C3 expression is associated with poorer survival outcomes and could serve as a prognostic marker for patient survival in this context (Figure 9).

## 3. Discussion

Potentially useful biomarkers for cancer that could be used for diagnosis, prognosis, or treatment are called CT antigens. Hoffman et al. devised the current CT gene classification approach [8]. A subset of human meiotic genes has been identified as comprising CT genes and exhibiting a highly limited cancer-specific signature based on an in silico pipeline [9,25]. This experiment identified specific primer sequences that effectively amplified members of the MAGE-C family. The results indicated that all three genes were significantly overexpressed (upregulated) in CC tissue samples, which was corroborated by qRT-PCR analysis. This was the case when the NC tissue samples were compared to the CC tissue samples. Although the number of CC patients analyzed is small, the data continuously showed that the CC tissue samples had greater expression levels of these genes than the NC tissue samples. As a result, their expression patterns suggest that these genes may be useful indicators for CC early identification. The activation mechanism of *MAGE-C* expressions among CC tissues is most likely connected to the hypomethylation of the cellular genome. For instance, in cancer cells demethylation contributes to the stimulation of various CT expressions [6,26].

The mRNA expressions of these three genes have been reported to be strictly testis-restricted [17,19,20,21]. In this study, however, these genes are overexpressed at significant levels in CC tissues compared to adjacent NC tissues. This finding is in line with one previous study showing that in patients with CC, CT genes such as *FTHL17*, *PRM2*, *CABYR*, *CPXCR1*, *ADAM29*, and *CABS1* were more highly expressed in CC patients than the adjacent NC specimens [26]. This suggests that genes that are generally overexpressed in CC compared to NC from the same region may be useful for future immunotherapeutic protocols. 

Based on the clinical data of research participants, the majority of overexpressions of *MAGE-C1*, *MAGE-C2*, and *MAGE-C3* were detected in advanced-stage CC tissue samples, which include 14 and 4 samples from stages II and III, respectively, as reported in Table 1. The results suggest that *MAGE-C1*, *MAGE-C2*, and *MAGE-C3* could have a biological role in the late stage of CC tissue. In the majority of cancers, the CT gene expression pattern is heterogeneous. Therefore, additional research on many cases and tumors is required to determine a relationship between CT gene expression and tumor stage.

Consistent with previous reports on different malignancies regarding *MAGE-C* family expressions, the expressions of *MAGE-C1* and *MAGE-C2* genes have been correlated with advanced stages of BC, multiple myeloma (MM), thyroid malignancies, and CC [16,24,27,28]. There has also been evidence of *MAGE-C2* expression at advanced stages of breast cancer [27] and esophageal squamous cell carcinoma [29]. In addition, *MAGE-C1* expression was found to be significantly correlated with tumor grade and to exhibit more aggressive behavior in breast and ovarian cancers, according to previous studies [30,31]. Frequent CT gene expressions have been identified as being associated with a higher tumor grade and more aggressive clinical features for various cancer forms, including melanoma and BC [10,32].

Previous studies identified that *MAGE-C1* and *MAGE-C2* genes were expressed in 71% and 29%, respectively, in patients with MM [27]. Previous studies showed that *MAGE-C2* was expressed most frequently in patients with hepatocellular carcinoma and non-small cell lung cancer but not expressed in the corresponding adjacent healthy tissue samples [33,34], which suggests that they could be used as a cancer biomarker. High *MAGE-C3* expression levels were observed in patients with ovarian cancer compared to normal ovary tissues [35]. These inconsistent findings could be attributed to population-specific differences or variations in sample types may account for these inconsistencies as well as using different primer sets (variations) in the physiology of the clinical samples. Our study focuses on a specific cohort of Saudi patients, and it is possible that the expression profiles of MAGE-C family genes vary across different populations. Ethnic and genetic diversity can contribute to variations in gene expression.

According to different studies, most cells exhibit epigenetically suppressed CT gene expression due to promoter region methylation. According to recent works, global hypomethylation is likely the cause of CT gene expression in CC cells [6,23]. Drugs that inhibit methylation, like 5-aza-2′-CdR, have been utilized to enhance the expression of these CT genes [26]. Studies have shown that the activation of CT gene expression occurs in various types of cancer cells when treated with drugs that disrupt DNA methylation [6,11,36,37]. Research has demonstrated that hypomethylating agents can activate a select group of X-CT genes, which play a critical role in regulating DNA methylation and silencing CT genes [6,11].

To determine whether the reduction of DNA methyltransferase could enhance the expression of *MAGE-C* genes transcripts, we treated early passage HCT116, Caco-2, MCF-7, and MCF-10A cells with 10 µM of 5-aza-2′-CdR for 72 h. We initially assessed the effect of 5-aza-2′-CdR on *DNMT1* expression levels. As shown in Figure 3, the results indicated that all cell types treated with 5-aza-2′-CdR exhibited reduced expression of the key methylation repair enzyme *DNMT1* when compared to the DMSO-treated control cells. Previous studies have also reported decreased levels of *DNMT1* expression across various cancer tissue types, supporting our findings [6,26,38]. Future research is needed to investigate whether treatment with 5-aza-2′-CdR influences the expression levels of other types of *DNMTs*.

Furthermore, the findings of the 5-aza-2′-CdR experiment demonstrated that the levels of *MAGE-C* family genes expression were activated in HCT116 Caco-2, MCF-7, and MCF-10A cells (Figure 4 and Figure 5). According to these findings, DNA hypomethylation is the mechanism that is linked to activating *MAGE-C1*, *MAGE-C2*, and *MAGE-C3* expressions in HCT116, Caco-2, MCF-7, and MCF-10A cells. This activation occurs through the demethylation of the promoter CpG islands of these cells. On multiple occasions, it has been established that CT genes are expressed in solid tumors [7,11,12,26]. These results confirmed that the increased expression levels of the *MAGE-C* genes in the CC and BC tissues are directly caused by a biological mechanism known as DNA hypomethylation.

Hypomethylation can be attributed to genomic instability, oncogene activation, and imprinting loss [39]. Tumor suppressor genes with specific promoter hypermethylation are silenced, leading to many characteristics of cancer, including resistance to antigrowth signals, prolonged angiogenesis, unrestricted replication potential, invasion of surrounding tissues, and metastasis. Past research has demonstrated that cancer patients with abnormal gene methylation resist chemotherapy due to inhibited apoptosis. This alteration is reversible because methylation affects gene regulation but not DNA sequences. If the hypermethylation in the promoter region of the silent tumor suppressor genes is eliminated, the genes may be re-expressed, potentially inhibiting tumor growth [40,41,42].

Previous studies have shown that the DNA methyltransferase inhibitor 5-aza-2′-deoxycytidine (5-aza-2′-CdR) can dramatically change the expression of *CTCFL*, also referred to as BORIS. It has been demonstrated that the transcriptional regulator CTCFL modifies the expression of several CT genes, which are frequently aberrantly activated in cancer cells but silent in normal tissues [43,44,45]. Furthermore, previous studies have highlighted the role of histone deacetylase (HDAC) inhibitors (HDACis) in cancer therapy. HDAC inhibitors, by blocking histone deacetylation, can promote a more relaxed chromatin structure, facilitating transcriptional activation of various genes, including CT genes. Given the interplay between DNA methylation and histone modifications, future research should explore the combined effects of DNA methyltransferase inhibitors like 5-aza-2′-CdR and HDAC inhibitors on CT gene expression. Investigating these epigenetic regulators will provide deeper insights into the transcriptional control of CT genes and their potential as therapeutic targets in cancer treatment [11,46]. For instance, the activation of numerous CT genes was observed in CC cells that were subjected to experimental treatment with trichostatin A (TSA), which is an HDAC inhibitor. These genes include *SCP2D1*, *CTAG1A*, *TKTL2*, *ACTRT1*, *MAGE-A4*, *MAGE-B1*, *FTHL17*, *PRM2*, *CABYR*, *CPXCR1*, *ADAM29*, and *CABS1* [6,26]. Therefore, more research is required to determine whether *MAGE-C* gene expression may be regulated by inhibiting histone deacetylation.

The analysis of TCGA samples indicates that MAGE-C1 and MAGE-C2 are significantly upregulated in specific cancer types, including skin cutaneous melanoma (SKCM) and lung adenocarcinoma (LUAD). Furthermore, MAGE-C3 exhibits pronounced expression in glioblastoma multiforme (GBM) and moderate expression in other malignancies. These genes can potentially serve as biomarkers for cancer diagnosis and surveillance, as evidenced by their differential expression patterns [47]. This study indicates that the upregulation of these genes in CC may be influenced by demethylation, as evidenced by the substantial hypomethylation of MAGE-C1, MAGE-C2, and MAGE-C3 promoters in primary tumor tissues compared to normal tissues. This epigenetic modification suggests that the methylation status of the promoter could be a valuable biomarker for COAD and provide valuable information regarding therapeutic strategies that target these genes [48]. The survival plots indicate that high expression of MAGE-C1 is associated with better overall survival (HR = 0.75, log-rank *p* = 0.015), suggesting a protective role. The high expression of MAGE-C2 shows a trend towards poorer survival (HR = 1.21), though it is not statistically significant (log-rank *p* = 0.081). The high expression of MAGE-C3 is significantly associated with poorer survival outcomes (HR = 1.46, log-rank *p* = 0.0053), indicating its potential as an unfavorable prognostic biomarker [24].

Our study focused on Saudi CC patients, a relatively homogenous population, which may explain why we observed elevated levels of *MAGE-C1*, *MAGE-C2*, and *MAGE-C3* that were not seen in the broader TCGA cohort. Other studies have shown similar population-specific variations in cancer biomarkers, suggesting that what holds true in one population may not directly apply to another. Therefore, our findings may reflect regional or genetic factors specific to the Saudi population, reinforcing the need for geographically targeted research in cancer biomarkers. Another factor contributing to the discrepancy may be the type of samples used. While our data were derived from pure tumor tissue samples obtained directly from CC patients, TCGA data are based on bulk tissue samples that may include contributions from surrounding stromal, immune, or normal cells. This heterogeneity in the sample composition may obscure the tumor-specific expression of MAGE-C genes. It is possible that in our carefully isolated tumor tissues, the expression of MAGE-C genes is more pronounced compared to bulk samples that dilute the tumor-specific signals. While our study highlights the potential for MAGE-C gene expression to serve as a biomarker for CC in Saudi patients, the lack of consistent findings in TCGA data suggests that the utility of these genes as universal biomarkers may be limited by population specificity or tissue sampling techniques. Therefore, we propose that the MAGE-C family genes, particularly in the context of hypomethylation, may be more relevant in specific subpopulations and require further validation in larger, more diverse cohorts.

Finally, there are a few limitations related to the current study. Initially, the results need to be confirmed with bigger samples of both males and females because this study included only 26 surgical samples, with 20 from males and 6 from females. Second, a lack of samples prevented the evaluation of the potential of MAGE-C genes’ protein levels. Third, even though we looked at the expression of three CT genes, *MAGE-C1*, *MAGE-C2*, and *MAGE-C3*, in CC tissues, more than 100 CT genes still need to be investigated.

## 4. Materials and Methods

### 4.1. Ethical Approval and Sample Collection

This study was conducted in full compliance with ethical standards and received official approval from the Ethics Committee of Al-Imam Muhammad Ibn Saud Islamic University. This project was granted ethical clearance under approval number HAPO-01-R-011, with the specific project identification being 56-2020. This ethics committee operates under the oversight of the institutional review board (IRB), ensuring that all aspects of this research adhered to national and international ethical guidelines for biomedical research involving human participants. None of the participants had undergone treatments such as chemotherapy or physical therapy prior to this study. A team of surgeons and pathologists conducted thorough evaluations using standard clinical, endoscopic, radio-graphic, and histological methods to diagnose cancer and assess patient eligibility for this study. All participants provided written informed consent and received a privacy statement outlining the measures taken to protect their personal information. Additionally, participants completed a self-administered questionnaire that collected data on their ages, family medical histories, personal medical histories, any allergic conditions, and lifestyle factors, including alcohol consumption and smoking habits.

This research involved the collection of 26 matched male and female participants, consisting of CC and NC tissue samples. All individuals diagnosed with CC were identified between December 2020 and September 2024.

### 4.2. Sources, Culturing Methods, and an Epigenetic Drug (5-Aza-2′-CdR) for Cancer Cell Lines

We used human cancer cell lines, especially HCT116 and Caco-2, as well as the breast cancer cell line MCF-7 in this study. King Saud University’s Dr. Bader Almutairi kindly provided the HCT116 and Caco-2 lines, while the ATCC was consulted for the acquisition of MCF-7. All cell lines were maintained in 37 °C incubators with DMEM (catalog number 61965026; Thermo Fisher Scientific, Waltham, MA, USA) and 10% FBS (catalog number A3160801; Thermo Fisher Scientific, Waltham, MA, USA) in a controlled atmosphere with 5% CO_2_. DMSO was utilized to dilute 5-aza-2′-CdR (Sigma: A3656) to achieve the required final concentration. Each of the three cell lines, HCT116, Caco-2, and MCF-7, was treated in two groups. The first group was exposed to 10 μM of 5-aza-CdR for 72 h, and the second group was exposed to DMSO for 72 h (as a negative control for 5-aza-CdR). The timing and dosing of the 5-aza-2′-CdR drug were determined by the findings of our recent publications [1,6,23].

### 4.3. Sources, Culturing Methods, and an Epigenetic Drug (5-Aza-2′-CdR) for NB Cell Lines

The cultivated cells were maintained in a medium composed of DMEM supplemented with 10% FBS, 2 mM glutamine, and a combination of antibiotics, including 100 IU/mL penicillin and 100 μg/mL streptomycin. The samples were stored in a humidified incubator at a temperature of 37 °C with a CO_2_ concentration of 5%. All growth factors were sourced from Sigma (St. Louis, MO, USA).

### 4.4. Extraction of RNA from NC, CC, and Cultured Cells

Total RNA isolation and purification were performed using the All-Prep DNA/RNA Mini Kit (80204; Qiagen, Hilden, Germany) to ensure high-quality RNA for downstream analysis. The process followed the manufacturer’s detailed instructions to maintain consistency and accuracy. For each sample, approximately 30 mg of both NC and CC tissue was carefully excised and transferred to clean, sterile Eppendorf tubes. In addition to the tissue samples, the total RNA was also isolated from cultured cells grown to a density of 5 × 10^6^ cells. The same All-Prep DNA/RNA Mini Kit was used, with all steps meticulously carried out according to the manufacturer’s protocol to ensure reliable and comparable RNA yields across both tissue and cell samples. After isolating RNAs, their concentrations were determined using the methods used in prior reports [6,7].

### 4.5. Synthesis of cDNA

For this process, 2000 ng of RNA from each sample was reverse-transcribed into cDNA using the High-Capacity cDNA Reverse Transcription Kit (4368814; Applied Biosystems, Hilden, Germany), following the manufacturer’s detailed protocol. The reverse transcription reaction was prepared by combining RNA with a reaction mix that included reverse transcriptase, random primers, dNTPs, and buffer. This reaction was then subjected to a thermal cycling program that consisted of an initial step at 25 °C for 10 min, followed by 37 °C for 120 min to allow reverse transcription to occur, and finally the enzyme was inactivated by heating at 85 °C for 5 min. Upon completion of the cDNA synthesis, the resulting cDNA was diluted at a ratio of 1:10 using nuclease-free water.

### 4.6. Designing RT-PCR Primers, Setting Up RT-PCR Reactions, and Performing Agarose Gel Electrophoresis of the Products

The RT-PCR primers used in this study were designed using a combination of manual selection and automated computational approaches, as detailed in previous literature [6,7]. The design process ensured that the primers were specific to the target gene sequences, optimizing for both efficiency and accuracy in amplification. All primers were produced by Macrogen (Macrogen Inc., Seoul, Republic of Korea) and subsequently diluted in nuclease-free water to achieve a final concentration of 10 μM (10 pmol/μL). This dilution was carefully prepared to ensure consistent results across all reactions. Table 3 outlines the RT-PCR primer sequences for each gene, alongside the expected amplicon sizes, providing a comprehensive guide to the genes targeted in this experiment. To assess the quality and integrity of the synthesized cDNA, amplification of the housekeeping gene *ACTB* was performed across all cDNA samples, including those derived from cancer, treated, and untreated tissues. This served as an internal control to confirm the success of cDNA synthesis and the overall viability of each sample. Additionally, to further validate the specificity of each MAGE-C primer, RT-PCR was conducted using cDNA synthesized from RNA extracted from human testis tissue (AM7972; Thermo Fisher Scientific) known to express MAGE-C genes. This verification step ensured that the designed primers were accurately targeting the desired MAGE-C sequences without off-target amplification.

For each gene, RT-PCR reactions were set up in a total reaction volume of 20 µL. Each reaction contained 8.4 µL of nuclease-free water, 0.8 µL of diluted cDNA (200 ng/µL), 0.8 µL of both forward and reverse primers (10 μM each), and 10 µL of BioMix Red (BIO-25006; BioLine, London, UK). These components were added to clean PCR tubes to minimize contamination, and the reactions were mixed thoroughly before beginning thermal cycling. The RT-PCR cycling included a pre-denaturation step for 5 min at 96 °C, followed by a 35 denaturation step for 30 s at 96 °C, annealing for 30 s at 58 °C, an extension step for 30 s at 72 °C, and a final extension step for 5 min at 72 °C. Gel electrophoresis was performed using 1× TBE buffer containing 1% agarose (A9539; Sigma-Aldrich) and 0.5 g/mL ethidium bromide (46067; Sigma), running at 100 voltage for 1 h to separate 8 μL of each PCR product. To verify the accuracy of the amplified product sizes, a 100 bp DNA molecular weight marker (N0467; New England BioLabs, Ipswich, MA, USA) was loaded alongside the samples, with 3 µL added to the gel. This allowed for precise comparison between the observed and expected sizes of the PCR products, ensuring the success of the amplification process.

### 4.7. Designing qRT-PCR Primers and Setting Up qRT-PCR Reactions

Every qRT-PCR primer set was manually designed using the optimal criteria in prior studies [6,7]. The primers were synthesized by Macrogen, a well-known commercial provider of oligonucleotides. The final working concentration of the primers was adjusted to 10 μM to achieve optimal amplification efficiency. The details of the primers, including their sequences and the expected sizes of the PCR amplicons, are provided in Table 4. These primers were carefully selected to ensure that they would generate specific and re-producible products for each target gene. For setting up the qRT-PCR reactions, we utilized a 96-well plate format, which allowed us to perform multiple reactions simultaneously. We followed the instructions from the manufacturer of the iTaq Universal SYBR Green (1725120; Bio-Rad) to ensure that the reagents were properly mixed and that the reaction conditions were optimal for real-time analysis. Each qRT-PCR reaction had a total volume of 10 µL, which included the following components: 2 µL of diluted cDNA (100 ng/µL), 5 µL of SYBR Green (which contains the necessary buffer, dNTPs, and SYBR Green dye for fluorescence detection), 0.5 µL of each forward and reverse primer (10 µM), and 2.5 µL of nuclease-free water.

The QuantStudio™ 7 Flex Real-Time PCR System (Applied Biosystems, Hercules, CA, USA) was used to run the qRT-PCR assays. This system allowed for a highly sensitive detection and accurate quantification of the gene expression levels. To ensure the reliability of our results, each sample was analyzed in triplicate, minimizing the potential for technical variability. In addition to these amplification cycles, a melting curve analysis was performed at the end of the qRT-PCR run.

### 4.8. Statistical Analysis

The SPSS statistical package was used for qRT-PCR analyses (ver.22; SPSS Inc., Chicago, IL, USA). In this study, all *p*-values (* p ≤ 0.05, ** p ≤ 0.01, *** p ≤ 0.001, and **** p ≤ 0.0001) were regarded as statistically significant.

### 4.9. In Silico Analysis

The expression data for patients diagnosed with colon adenocarcinoma were retrieved from The Cancer Genome Atlas (TCGA) database using the cBioPortal platform [49] (https://www.cbioportal.org/, accessed on 17 July 2024). RNA-Seq expression data from the TCGA-RNAseq project were analyzed, encompassing samples from 313 patients diagnosed with colon adenocarcinoma. The data not only included RNA-Seq data (CPTAC-RNAseq) but also proteomic data (CPTAC-proteome), which allowed for a thorough investigation of both transcriptomic and protein-level expressions. In addition to cBioPortal, we utilized the UALCAN web resource (http://ualcan.path.uab.edu/analysis.html, accessed on 17 July 2024), for further analysis [50]. UALCAN is an accessible, user-friendly platform that facilitates the exploration of publicly available cancer-related transcriptome data, specifically from the TCGA and MET500 cohorts [50].

### 4.10. Survival Analysis

The Kaplan–Meier plotter tool was utilized to conduct survival analysis for colon adenocarcinoma [51]. The Kaplan–Meier plotter is an advanced online platform designed to evaluate the association between gene expression and patient survival across multiple cancer types. The Kaplan–Meier plotter tool, accessible at http://kmplot.com/analysis/ (accessed on 17 July 2024) was utilized to perform the survival analysis. As of 15 July 2024, the platform integrates expression data from over 35,000 samples across 21 different tumor types, including uterine corpus. Kaplan–Meier survival curves were plotted to visualize survival differences between high- and low-expression groups. Log-rank tests were used to assess the statistical significance of differences in survival curves. *p*-values obtained from the log-rank test and FDR-adjusted p-values were used to determine the significance of the association between gene expression and survival outcomes. The results were interpreted in the context of their potential biological and clinical relevance to uterine corpus endometrial carcinoma. Significant genes with notable differences in survival outcomes were further analyzed for their potential as biomarkers or therapeutic targets. Survival probability over time for both the low- and high-expression groups were estimated using Kaplan–Meier survival curves. Gene expression and clinical data were acquired from publicly accessible cancer datasets, such as TCGA or Gene Expression Omnibus (GEO) [52]. The dataset contained the gene expression levels of MAGE-C (206609_at) and the accompanying survival statistics for cancer patients. The log-rank *p*-value was utilized to evaluate the statistical significance of the observed disparities in survival. A p-value less than 0.05 was deemed statistically significant, suggesting a notable disparity in survival rates between the high- and low-expression groups. The hazard ratio (HR) and its 95% confidence interval (CI) offered valuable information regarding the relative risk of mortality linked to elevated MAGE-C expression. An HR less than one indicates a survival benefit for the group with high expression, whereas an HR greater than one indicates a higher risk of death.

## 5. Conclusions

This study indicates that elevated expression levels of *MAGE-C* family genes hold potential as biomarkers for CC, primarily due to their involvement in the demethylation process. While protein levels of these genes have yet to be assessed, our results imply that they may act as cancer-specific markers for the early identification of populations at risk for CC. Additionally, in vitro experiments demonstrated that the expression of the examined *MAGE-C* genes can be induced in cell lines from CC, BC, and NB through treatment with the agent 5-aza-2′-CdR. These findings suggest that 5-aza-2′-CdR is a crucial regulator of *MAGE-C* gene expression, as evidenced by the observed decrease in *DNMT1* expression. The upregulation and hypomethylation of these genes in tumors highlight their potential as biomarkers for cancer diagnosis and prognosis. Additionally, the differential survival outcomes associated with their expression levels suggest that targeting MAGE-C genes could improve therapeutic strategies. Furthermore, the hypomethylated process is critical for the transcriptional activation of *MAGE-C* genes and may be included in future cancer immunotherapies. Future studies should involve larger, more diverse patient cohorts and explore additional epigenetic mechanisms. Despite these limitations, our results provide valuable insights into the potential of MAGE-C genes as targets for the early detection of and therapeutic intervention in CC.

## Figures and Tables

**Table 1:** Subjects with CC and NC: general clinical features.

Variables	CC (*n*%)			NC (*n*%)		
Male participants	20 (100%)			20 (100%)		
Mean age (min–max)	57 (24–79)			57 (24–79)		
Below 57	8 (40%)			8 (40%)		
Above 57	12 (60%)			12 (60%)		
Female participants	6 (100%)			6 (100%)		
Mean age (min–max)	59 (30–80)			59 (30–80)		
Below 59	3 (50%)			3 (50%)		
Above 59	3 (50%)			3 (50%)		
	Cancer stage in CC					
Stage of CC	I		II		III	
CC patient sample	2–5, 10, 13, 20		6–9, 12, 16–19, 21–26		1, 11, 14–15	

Abbreviations: CC: colon cancer; NC: normal colon; n: number of samples.

**Table 2:** The cell proliferation of cancer and normal cell lines treated with 10 µM of 5-aza-2′-CdR.

Cell Type	Exposure Duration of Cells to 5-Aza-2′-CdR		
	Day 1 (%)	Day 2 (%)	Day 3 (%)
HCT116	98	95	91
Caco-2	98	94	90
MCF-7	96	92	88
MCF-10A	99	95	90

**Table 3:** RT-PCR primer sequences designed for the detection of *MAGE-C* genes and their anticipated product sizes.

Genes	Accession Number	Category	Primer Sequences (5′→3′)	Ta	Product Size (bp)
*ACTB*	NM_001101.5	Forward Reverse	AGAAAATCTGGCACCACACC AGGAAGGAAGGCTGGAAGAG	58 °C	553
*MAGE-C1*	NM_005462.5	Forward Reverse	GTTCCAAGTCTTCCTGAGTG TGGAGAGAAGACTGGAAGTC	58 °C	675
*MAGE-C2*	NM_016249.4	Forward Reverse	CATGGAGCTCATTCAGTGAG CCGATACTCCAGGTAATGTC	58 °C	539
*MAGE-C3*	NM_138702.1	Forward Reverse	CAGTGAAGAGGAGGATACAG GACACAGCTGCCCTTTATGA	58 °C	391

Abbreviations: RT-PCR: reverse transcription polymerase chain reaction; A: adenine; T: thymine; C: cytosine; G: guanine; Ta: annealing temperature; bp: base pair.

**Table 4:** qRT-PCR primer sequences designed for the detection of *MAGE-C1*, *MAGE-C2*, *MAGE-C3*, and *DNMT1* genes with their anticipated product sizes.

Genes	Category	Primer Sequences (5′→3′)	Ta	Product Size (bp)
*GAPDH*	Forward Reverse	GGGAAGCTTGTCATCAATGG GAGATGATGACCCTTTTGGC	58 °C	173
*MAGE-C1*	Forward Reverse	CAGATTCCTATGACCTCCTC CAGTAAAGTGGAGGAGAAGG	58 °C	144
*MAGE-C2*	Forward Reverse	CATGGAGCTCATTCAGTGAG CTGCTTCGTATTTGAGGAGC	58 °C	153
*MAGE-C3*	Forward Reverse	CAGTGAAGAGGAGGATACAG AGCATCTCTGCCTTTGTGAC	58 °C	150
*DNMT1*	Forward Reverse	GTAAAGCCTGCAAGGACATG CATCGACTTCCTCATCGTCA	58 °C	120

Abbreviations: qRT-PCR: quantitative RT-PCR; A: adenine; T: thymine; C: cytosine; G: guanine; Ta: annealing temperature; bp: base pair.

## Data Availability

All data generated or analyzed during this study are included in this published article.
